# Metastatic Renal Cell Carcinoma Masquerading as a Laryngeal Tumor: A Case Report

**DOI:** 10.7759/cureus.39229

**Published:** 2023-05-19

**Authors:** Sk Soel Ahmed, Sandip Kumar Barik, Amit Kumar Adhya, Deepak Kumar Das, Archishnu Vedanta Parida, Priyanka Mukherjee, Saroj Kumar Das Majumdar, Dillip Kumar Parida

**Affiliations:** 1 Department of Radiation Oncology, All India Institute of Medical Sciences, Bhubaneswar, Bhubaneswar, IND; 2 Department of Pathology and Laboratory Medicine, All India Institute of Medical Sciences, Bhubaneswar, Bhubaneswar, IND

**Keywords:** laryngeal tumor, case report, renal cell carcinoma (rcc), metastatic renal cell carcinoma, tumor

## Abstract

Renal cell carcinoma (RCC) commonly metastasizes to various organs such as the lungs, liver, bones, and brain. However, isolated metastases to the head and neck region, especially the larynx, are very rare. This report presents a case of laryngeal growth that was eventually confirmed to be a metastatic deposit from an undiagnosed RCC. We report a case of a 66-year-old male who presented to the clinic with painless neck swelling and a change in voice. The scan showed a soft tissue mass in the thyroid cartilage. Histopathology of the resected laryngeal tumor confirmed metastatic clear cell carcinoma. A metastatic workup revealed a renal mass, and the patient underwent laparoscopic adrenal-sparing left cytoreductive nephrectomy. The histopathological examination established the diagnosis of clear cell RCC. Subsequently, the patient was treated with pembrolizumab and lenvatinib. Follow-up imaging showed no residual or recurrent lesions. This case highlights the rarity of laryngeal metastasis from RCC and the importance of an accurate diagnosis through advanced imaging and histopathological examination.

## Introduction

Renal cell carcinoma (RCC) is the world's 12th most common malignant neoplasm, excluding blood cancers [1]. At presentation, around 30% of RCC patients have metastatic disease, whereas 70% become metastatic after diagnosis. The lungs, liver, bones, suprarenal glands, pleura, brain, and soft tissues are among the common sites of metastasis for RCC [2]. However, isolated RCC metastases to the head and neck region, particularly the larynx, are extremely rare [3].RCC is the second most common cancer to metastasize to the larynx after skin cancer [4], and secondary tumors of the larynx account for only 0.09% to 0.4% of all laryngeal tumors [5]. As a malignant disease, RCC is a great imitator with an erratic clinical course and biological behavior. This report describes a case of laryngeal growth that was later confirmed to be a metastatic deposit from an unrecognized RCC.

## Case presentation

A 66-year-old male smoker presented with a painless swelling that gradually increased in size over the left side of the upper neck region for the past three years. He had a change in voice for the last month, which was not relieved with conservative treatment. His medical history revealed type 2 diabetes mellitus, managed with injection insulin for three years. On examination, he was noted to have a mass measuring 2x2 cm on the left side of the neck, which was firm in consistency. Oral cavity and systemic examination of the chest, abdomen, and heart were normal. His blood tests were within the standard limit, including complete blood count, kidney function test, and liver function test.

**Figure 1:**
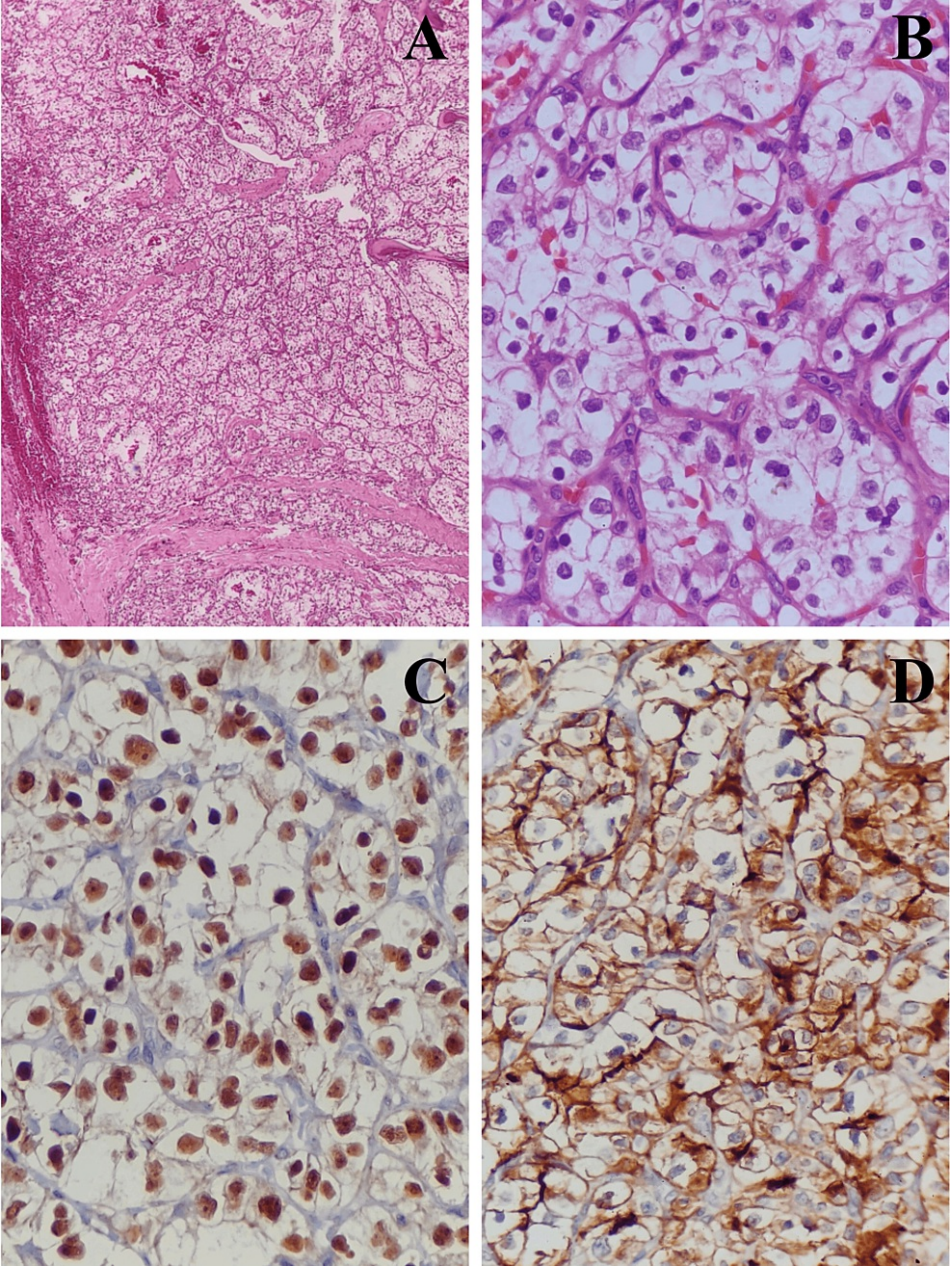
(A) The tumor is composed of nests of tumor cells separated by thin fibrovascular septa. The tumor is seen infiltrating the hyoid bone (H&E stain, 100x magnification). (B) The tumor cells are polygonal with abundant clear cytoplasm and centrally located nuclei (H&E stain, 400x magnification). (C) The tumor cells are positive for PAX 8 (IHC, 400x magnification). (D) The tumor cells are positive for CD 10 (IHC, 400x magnification). H&E, hematoxylin and eosin; IHC, immunohistochemistry

Because of the clinical and physical examination, a contrast-enhanced magnetic resonance imaging (CE-MRI) scan of the face and neck region was obtained, which revealed a well-circumscribed solid 2.8x2.0x2.7 cm soft tissue mass lesion with an epicenter in the left thyroid cartilage bulging into extralaryngeal and paraglottic space without eroding cricoid cartilage. Fine needle aspiration cytology of the mass lesion was performed, and it was reported as suspicious of malignancy. Direct laryngoscopy revealed normal vocal cord movement. No obvious abnormality was detected in the contrast-enhanced computed tomography (CECT) of the thorax. Excision of the laryngeal cartilage tumor with reconstruction was performed as the patient showed unwillingness for laryngectomy. Post-operative histopathology (HPE) of the resected laryngeal tumor specimen suggested metastatic clear cell carcinoma. Immunohistochemical staining was positive for CD-10 and PAX-8 and negative for p40, CK-7, and CK-20 (Figures 1A-1D).

**Figure 2:**
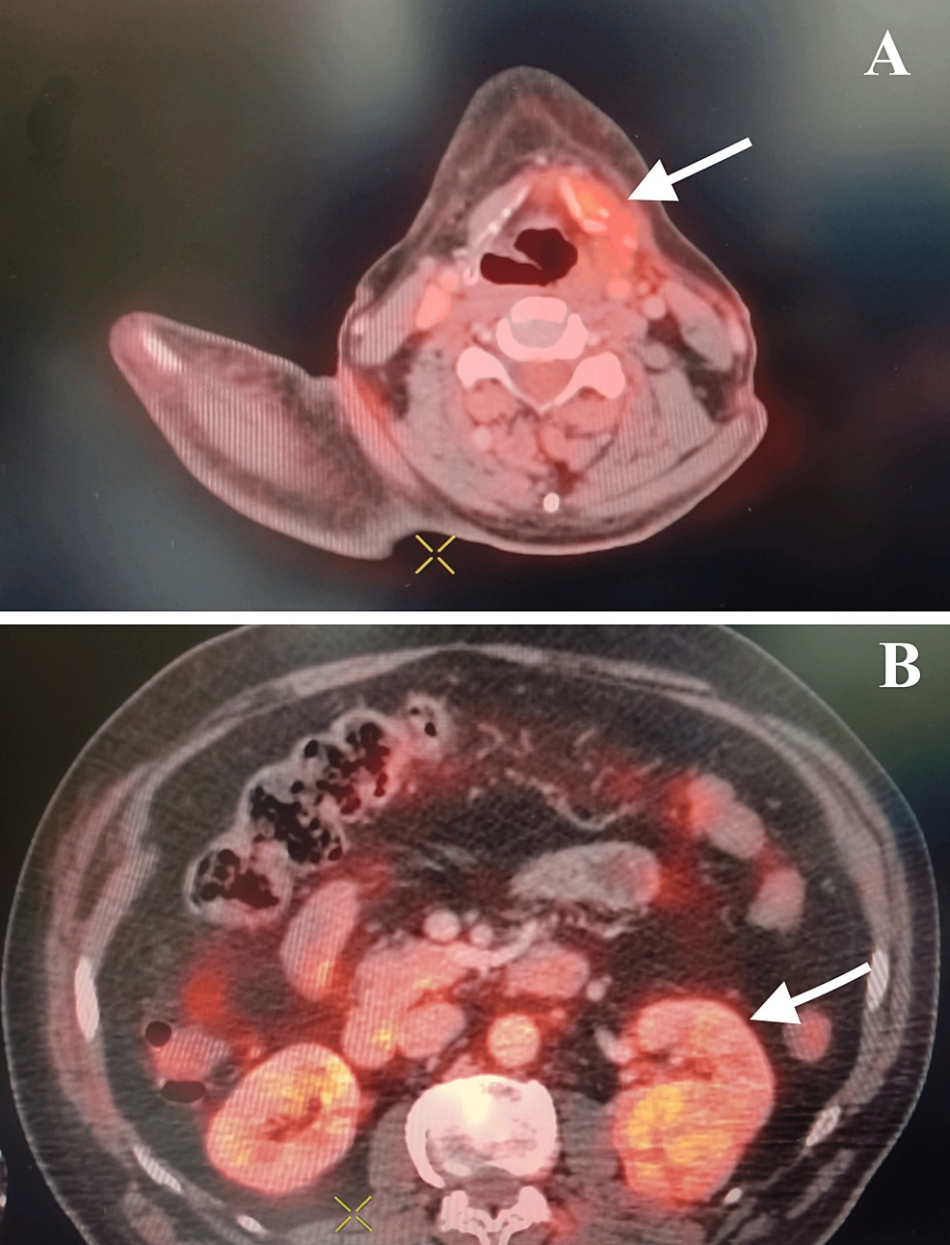
(A) An 18F-FDG PET-CT scan showing thickening with FDG uptake in thyroid cartilage and (B) in the left kidney (white arrows). FDG PET-CT, fluorodeoxyglucose positron emission tomography-computed tomography

An 18F-fluorodeoxyglucose positron emission tomography-computed tomography (FDG PET-CT) scan was performed to look out for the primary tumor. It showed mildly FDG avid 3.4x2.8 cm soft tissue mass in the lower pole of the left kidney and a heterogeneously FDG avid 1.2x1.3 cm soft tissue thickening in the left lamina of the thyroid cartilage (Figure 2).

The patient underwent laparoscopic adrenal-sparing left cytoreductive nephrectomy. Histological examination of the surgical specimen confirmed the diagnosis of clear cell RCC of WHO/ISUP (World Health Organization/International Society of Urologic Pathologists) grade 2. The pathological tumor, node, and metastasis (TNM) staging was pT1aNxM1. The patient was subsequently treated with pembrolizumab and lenvatinib. After four months of treatment, the PET-CT scan did not reveal any residual or recurrent lesion, and the patient is currently asymptomatic for the disease.

## Discussion

RCC is the 12th most common malignant neoplasm worldwide, excluding blood cancers, accounting for around 2.4% of all adult malignancies. Early detection of RCC is associated with a five-year survival of 93%; however, the metastatic disease has a poor five-year survival of 12% [1]. Due to a lack of reporting, the incidence of RCC is comparatively lower in Asian regions, particularly in India [6].

RCC is a group of tumors that originate from different parts of the nephron, with clear cell carcinoma being the most common subtype (75%), followed by papillary carcinoma (15%), chromophobe carcinoma (5%), and other subtypes making up less than 5%. Risk factors include males with higher age groups, hypertension, cigarette smoking, and excess body weight. Around 30% of patients with RCC are found to have metastasis at presentation. The lungs (50-60%), liver (30-40%), and bones (30-40%) are the most frequently affected sites of metastasis in RCC, along with the suprarenal glands, pleura, brain, and soft tissues. Metastasis to the head and neck region is less prevalent than in other sites (around 8-15 % of all metastasis from RCC). Head and neck sites are neck nodes (48%), para nasal sinus (34%), thyroid gland (14%), skull bone (10%), parotid (5%), tongue (5%), and facial skin (5%) [2].

Secondary laryngeal tumors comprise 0.09-0.4% of all laryngeal tumors [5]. Distal tumors rarely metastasize to the larynx because it is terminally located in the lymphovascular circulation. After skin cancer, RCC is the second most frequent type of cancer to metastasize to the larynx, with 31 cases reported till now. Metastasis to the larynx occurs via an anterograde or retrograde pathway. The anterograde pathway includes a hematogenous route. Retrograde spread occurs via Batson's venous plexus [4].

Clinically, secondary laryngeal tumors do not differ from those of primary tumors except for hemoptysis. It is a standard indicator of laryngeal metastasis from RCC because these tumors have high vascular stroma [7].

Histochemically, RCC usually stains positive for glycogen and lipid [7]. The presence of CD-10 and vimentin supports the diagnosis of RCC [5]. However, it is essential to note that clear cell or hypernephroid variety may also be found in the squamous, mucoepidermoid, adenosquamous, and acinar cell carcinomas, along with the primary clear cell carcinoma of the larynx [8].

The management of metastatic RCC is determined by the extent of local and systemic disease spread and the patient's overall prognosis. Treatment options include metastasectomy, palliative chemotherapy, radiotherapy, and the best supportive care [9]. Surgery of the metastatic lesion improves survival. It was seen that metastasis developing after nephrectomy was associated with improved survival than metastasis at diagnosis. Although RCC has been described as a radio-resistant tumor, studies have shown an excellent response to chemotherapy and radiotherapy [10]. Survival outcomes have improved in the past decade using targeted therapy and immunotherapy [11].

RCC is commonly called the "great imitator" because it tends to present as a metastatic disease, causing symptoms unrelated to the kidney. Since the classical clinical symptoms (hematuria, lumbar mass, and abdominal pain) come late during the condition, it can lead to diagnostic confusion. Our case entirely agrees with this observation, where the patient presented with neck swelling and hoarseness of voice. Our report adds to the limited number of cases documented in the literature on RCC metastasizing to the larynx. This is also the first case to be reported from India on undiagnosed RCC presenting as metastasis to the larynx.

## Conclusions

Laryngeal metastasis from RCC as a solitary lesion is extremely rare. A detailed history, careful clinical examination, and advanced imaging techniques can lead to early diagnosis and treatment. This case highlights the role of an excellent histopathological examination that can undermine any such hidden diagnosis, which is of utmost importance for appropriate treatment. A multidisciplinary approach should be administered to improve the quality of life while minimizing morbidity. Immunotherapy improves the poor prognosis and survival in metastatic RCC.
